# Hybrid therapy for acute upper limb ischaemia with thoracic outlet syndrome: a case report

**DOI:** 10.1093/ehjcr/ytag280

**Published:** 2026-04-21

**Authors:** Eiji Koyama, Kazuki Tobita, Motoaki Kai, Hirokazu Mityashita, Shigeru Saito

**Affiliations:** Department of Cardiology, Shonan Kamakura General Hospital, 1370-1 Okamoto, Kamakura, Kanagawa 247-8533, Japan; Department of Cardiology, Shonan Kamakura General Hospital, 1370-1 Okamoto, Kamakura, Kanagawa 247-8533, Japan; Department of Cardiology, Shonan Kamakura General Hospital, 1370-1 Okamoto, Kamakura, Kanagawa 247-8533, Japan; Department of Cardiology, Shonan Kamakura General Hospital, 1370-1 Okamoto, Kamakura, Kanagawa 247-8533, Japan; Department of Cardiology, Shonan Kamakura General Hospital, 1370-1 Okamoto, Kamakura, Kanagawa 247-8533, Japan

**Keywords:** Acute limb ischaemia, Arterial thoracic outlet syndrome, Hybrid therapy, Endovascular treatment, Stent graft, Case report

## Abstract

**Background:**

Arterial thoracic outlet syndrome (ATOS) is characterized by upper extremity ischaemia or aneurysm-like disease caused by external compression of the subclavian or axillary arteries at the thoracic outlet. Arterial thoracic outlet syndrome is the least common type of thoracic outlet syndrome (TOS), accounting for 1%–2% of all TOS cases. Acute limb ischaemia (ALI) is a life-threatening condition requiring urgent assessment and management. Although ALI most commonly affects the lower limb, 20% of cases involve the upper limb. The first-line treatment for ALI is surgical thrombectomy; however, some reports have found endovascular treatment to be effective.

**Case summary:**

A 45-year-old man complained of rest pain and paraesthesia in the left arm for the past week. Physical examination revealed coldness and pallor of the left upper limb and absence of the brachial pulse. Computed tomography revealed pseudarthrosis due to left cervical ribs, aneurysmal change of the left subclavian artery with thrombus, and distal occlusion beyond the brachial artery. Electrocardiography showed a normal sinus rhythm, and echocardiography showed no thrombus in the left ventricle. We diagnosed acute upper limb ischaemia due to subclavian artery aneurysms with TOS. He underwent emergent surgical thrombectomy via the left brachial artery. Surgical resection of the cervical and first rib was performed 1 month later, and endovascular treatment with a stent graft was performed for the aneurysmal change 3 months later.

**Discussion:**

We report a rare case of TOS with aneurysmal change and thrombosis. Arterial thoracic outlet syndrome should be considered in acute upper limb ischaemia when the embolic cause is unknown.

Learning points

- Approximately 20% of acute limb ischaemia cases occur in the upper limb, and over 50% of these are due to cardiac aetiologies such as atrial fibrillation.
- Atrial thoracic outlet syndrome should be considered in acute upper limb ischaemia when the cause of embolism is unknown.

## Introduction

Thoracic outlet syndrome (TOS) is a complex disorder characterized by signs and symptoms resulting from compression of the brachial plexus or subclavian vessels supplying the upper limb. Arterial thoracic outlet syndrome (ATOS) is a subset of TOS that presents with upper extremity ischaemia or aneurysm-like disease caused by external compression of the subclavian or axillary arteries at the thoracic outlet.^1^ Arterial thoracic outlet syndrome accounts for less than 1% of all TOS cases, in contrast to the more common neurogenic and venous subtypes.^2^ It is also a potential aetiology of acute upper limb ischaemia and is frequently associated with diagnostic challenges.

Acute limb ischaemia (ALI) is a life-threatening condition requiring urgent assessment and management. Approximately 20% of ALI cases occur in the upper limb, and over 50% of these are due to cardiac aetiologies such as atrial fibrillation.^3,4^ The first-line treatment for ALI is surgical intervention, although endovascular treatments (EVTs) may be employed in selected cases. Endovascular treatment options include thrombo-aspiration, catheter-directed thrombolysis (CDT), and angioplasty with thrombolytic agents. In our region, urokinase is the only insurance-covered thrombolytic agent approved for ALI treatment; however, it is currently unavailable due to manufacturing issues. Given the limited number of reported cases, there is no clearly established treatment strategy for upper limb ALI in current clinical guidelines.

## Summary figure

**Figure:**
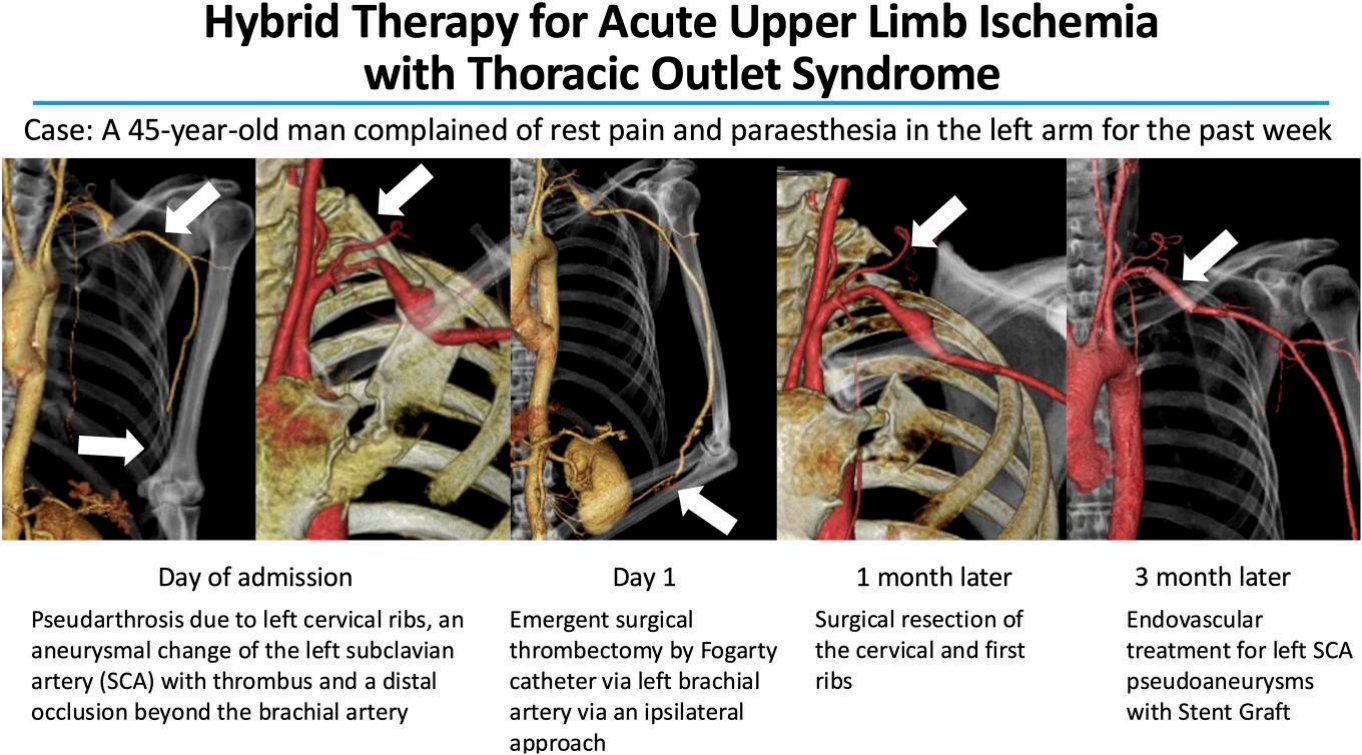


## Case presentation

A 45-year-old man complained of rest pain and paraesthesia in the left arm for the past week. He had no past medical history. His height was 175 cm, and body weight was 71 kg. Physical examination revealed coldness and pallor of the left upper limb and absence of the brachial pulse.

**Figure 1:**
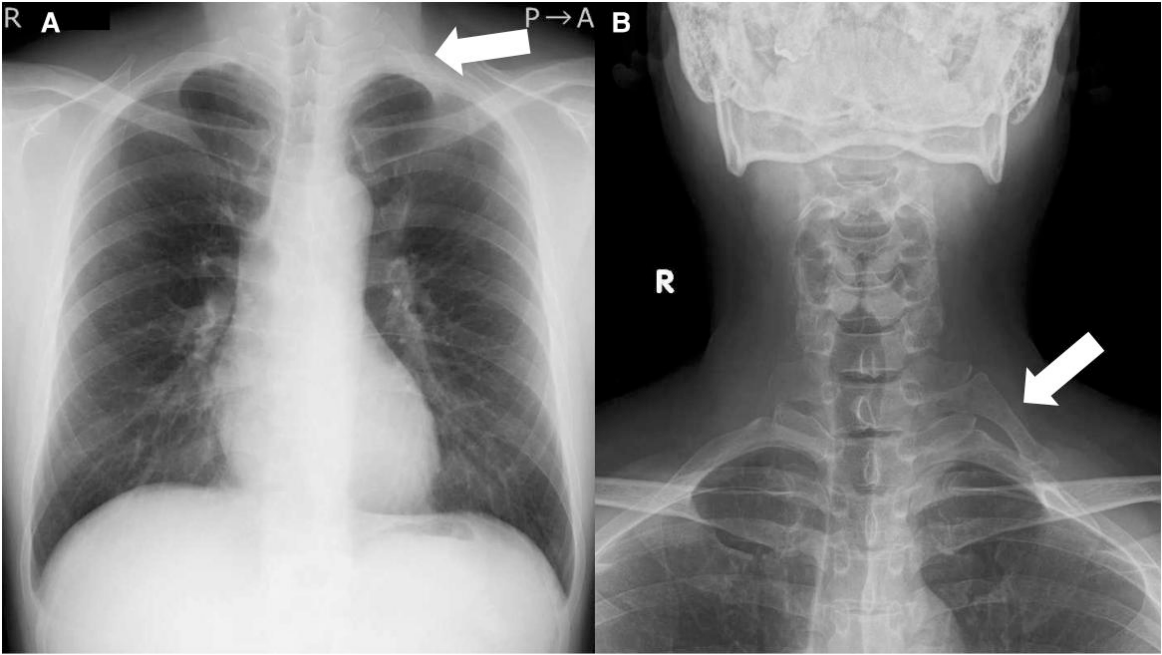
Chest (*A*) and cervical spine (*B*) radiography revealed a cervical rib on the left side (white arrows).

**Figure 2:**
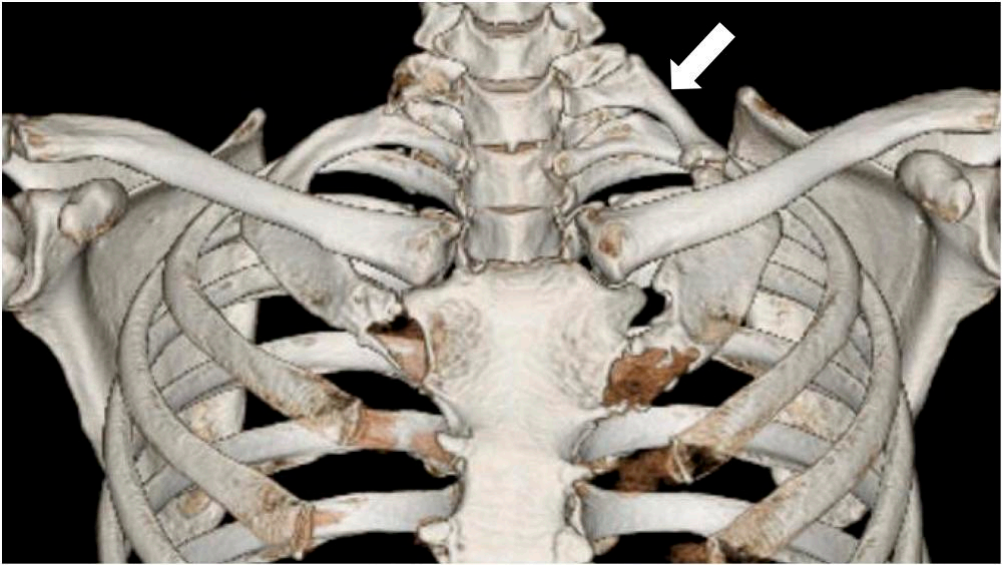
Computed tomography revealed pseudarthrosis due to left cervical ribs (white arrow).

**Figure 3:**
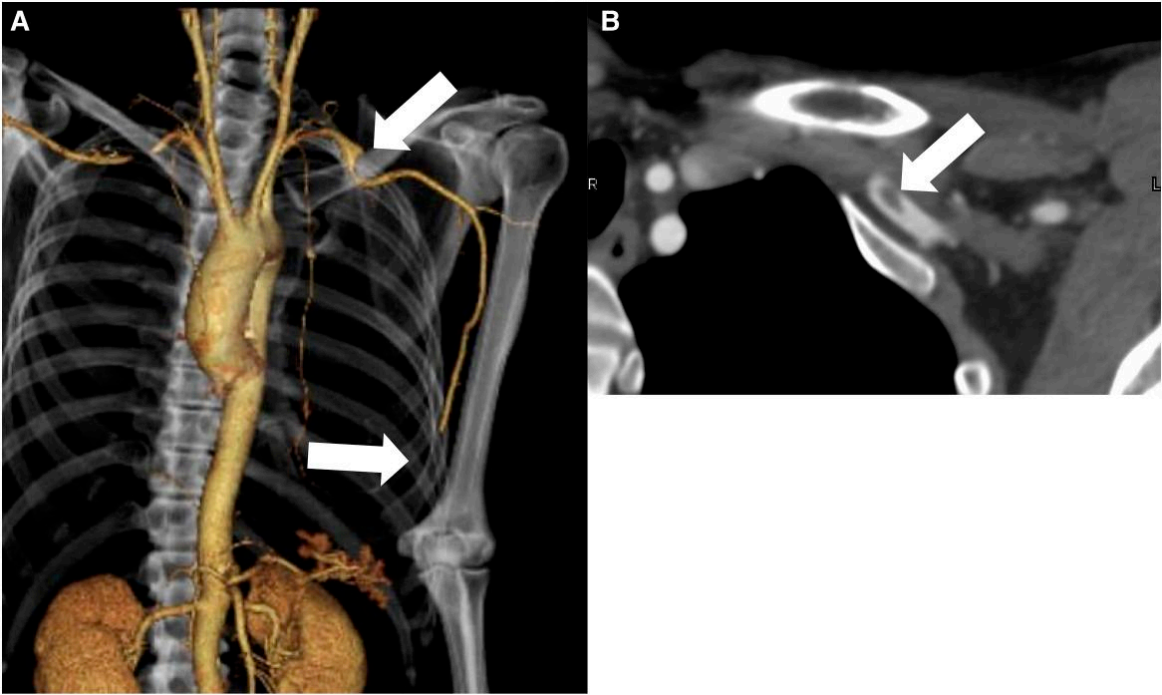
Contrast-enhanced computed tomography angiography revealed an aneurysmal change of the left subclavian artery with thrombus and a distal occlusion beyond the brachial artery.

Chest and cervical spine radiography revealed a cervical rib on the left side (*Figure 1*). Computed tomography (CT) showed pseudarthrosis due to left cervical ribs (*Figure 2*), aneurysmal change of the left subclavian artery (SCA) with thrombus, and distal occlusion beyond the brachial artery (*Figure 3A* and *B*). Electrocardiography revealed normal sinus rhythm, and echocardiography did not confirm thrombus in the left ventricle. The pre-operative Society for Vascular Surgery classification was Grade 2b.^5^ We diagnosed acute upper limb ischaemia due to SCA aneurysms with TOS.

He underwent urgent surgical thrombectomy using a Fogarty catheter via the left brachial artery. After surgery, axillary artery flow and his symptoms improved, and edoxaban (60 mg orally) was initiated. He was discharged from the hospital on Day 5 and underwent surgical resection of the cervical and first rib 1 month later at another hospital.

**Figure 4:**
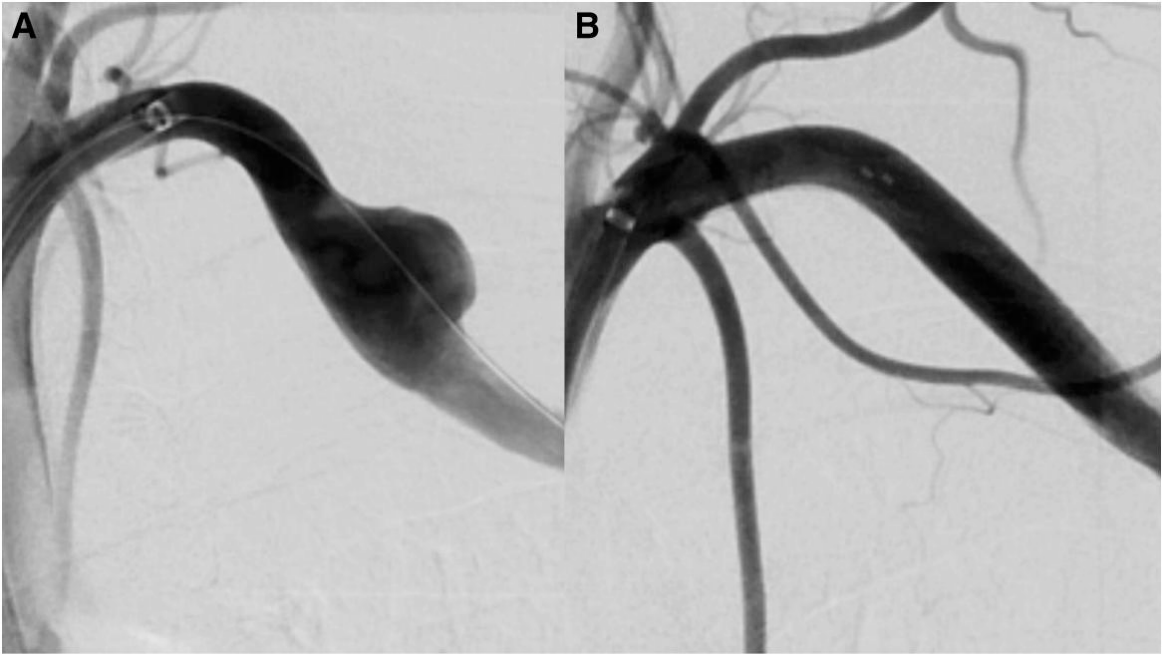
(*A*) Digital subtraction angiography revealed aneurysmal dilatation of the left subclavian artery. (*B*) Digital subtraction angiography demonstrated complete exclusion of the subclavian artery pseudoaneurysm.

Three months later, elective EVT for left SCA pseudoaneurysms was performed via the left common femoral artery using a 7 Fr Parent Cross (Medikit, Tokyo, Japan). Digital subtraction angiography revealed aneurysmal dilatation of the left SCA (*Figure 4A*). Lumen size and vascular structure disruption were assessed using intravascular ultrasound imaging, and an 8.0 × 50 mm VIABAHN stent graft (W.L. Gore & Associates, USA) was deployed across the left SCA pseudoaneurysm and post-dilated with an 8.0 mm semi-compliant balloon. The final angiogram showed complete exclusion of the SCA pseudoaneurysm (*Figure 4B*). He was discharged from the hospital and remained free of complications during the following 6 months.

## Discussion

Thoracic outlet syndrome is generally described as a group of symptoms, including arm pain, paraesthesia, and weakness, resulting from compression of the neurovascular bundle at the thoracic outlet. Approximately 2% of the North American population experiences symptoms of TOS, most commonly in women.^6^ Thoracic outlet syndrome is typically classified as neurogenic, venous, or arterial. Neurogenic TOS is by far the most common, accounting for about 95% of cases. Vascular forms are rare, representing 4% with venous aetiology and 1% with arterial aetiology.

Acute limb ischaemia is a life-threatening condition, with approximately 20% of cases occurring in the upper limb.^3^ More than 50% of upper limb ALI cases are caused by cardiac aetiologies such as atrial fibrillation.^4^ Endovascular treatment can be used for ALI in selected cases with advances in technology. The key component of EVT is thrombolytic therapy; however, urokinase, the only insurance-covered thrombolytic agent in our region, has been unavailable due to manufacturing issues. In this case, thrombo-aspiration and angioplasty were unlikely to restore adequate vessel flow because of a large thrombus burden, and surgical management was therefore selected.

The management of ALI due to ATOS has been described by Pitcher *et al.*^2^ Initial treatment involves anticoagulation, with additional interventions such as brachial artery thromboembolectomy (BAT) or CDT considered depending on the clinical presentation. Definitive management of ATOS includes rib resection and arterial repair, which are recommended during the index hospitalization if the patient’s condition allows. In this case, BAT was performed by cardiovascular surgeons as part of the acute management. Because surgical treatment for ATOS could not be performed at our institution, the patient underwent rib resection at another hospital 1 month later. The SCA pseudoaneurysm was subsequently treated electively with EVT, and the patient remains under observation.

This case represents acute upper limb arterial occlusion due to a non-cardiogenic embolism. Therefore, identifying the underlying aetiology was essential. Computed tomography revealed a cervical rib and a pseudoaneurysm of the SCA with thrombus, strongly suggesting ATOS as the cause.

## Lead author biography


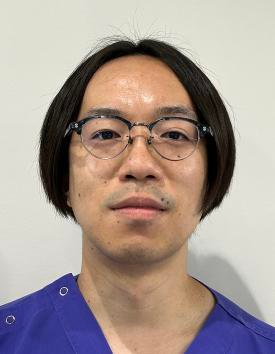
Eiji Koyama is a cardiac intervention specialist who works for Shonan Kamakura General Hospital in Japan. He received his medical degree at the Nihon University in 2017.

## Data Availability

The datasets used and/or analysed during the current study are available from the corresponding author upon reasonable request.
